# Room-temperature excitonic emission with a phonon replica from graphene nanosheets deposited on Ni-nanocrystallites/Si-nanoporous pillar array

**DOI:** 10.1098/rsos.172238

**Published:** 2018-08-15

**Authors:** Zhaojun Tang, Tingting Xu, Sen Li, Zhifeng Shi, Xinjian Li

**Affiliations:** 1 Department of Physics and Laboratory of Material Physics, Zhengzhou University, Zhengzhou 450001, People's Republic of China; 2 Electrical Engineering Department, Zhengzhou Business Technician Institute, Zhengzhou 450100, People's Republic of China

**Keywords:** graphene nanosheet, photoluminescence, excitonic emission, phonon replica

## Abstract

Graphene nanosheets (GNSs) were grown on a Si nanoporous pillar array (Si-NPA) via chemical vapour deposition, using a thin layer of pre-deposited Ni nanocrystallites as catalyst. GNSs were determined to be of high quality and good dispersivity, with a typical diameter size of 15 × 8 nm. Light absorption measurements showed that GNSs had an absorption band edge at 3.3 eV. They also showed sharp and regular excitonic emitting peaks in the ultraviolet and visible region (2.06–3.6 eV). Moreover, phonon replicas with long-term stability appeared with the excitonic peaks at room temperature. Temperature-dependent photoluminescence from the GNSs revealed that the excitonic emission derived from free and bound excitonic recombination. A physical model based on band energy theory was constructed to analyse the carrier transport of GNSs. The Ni nanocrystallites on Si-NPA, which acted as a metal-enhanced fluorescence substrate, were supposed to accelerate the excitonic recombination of GNSs and enhanced the measured emission intensity. Results of this study would be valuable in determining the luminescence mechanism of GNSs and could be applied in real-world optoelectronic devices.

## 1. Introduction

Graphene nanostructures can be engineered to emit photoluminescence (PL) over the visible region, even extending into the near infrared region [1–3]. PL from graphene nanostructures has different origins, such as the recombination of free excitons, quantum confinement or edge effects [4,5]. Excitonic effects play a critical role in the optical properties of graphene [6–8]. In nanomaterials, carriers are geometrically confined, which typically leads to fast recombination of the excitons before non-radiative recombination can occur. This condition results in a high radiative recombination probability and consequently strong PL. For example, the excitonic emission is responsible for the high efficiency of LEDs based on In*_x_*Ga_1−*x*_N nanorods, nanowire or multiple 2D quantum wells [9–11]. Therefore, the creation of nanostructures from graphene may be a promising route for realizing bright PL from this material.

The strong interaction between light and metallic nanostructures leads to the generation of surface plasmons and local enhancement of electromagnetic fields. Excited fluorophores can strongly interact with surface plasmons, and result in significant improvements in the spectral properties of fluorescent probes. These improvements include increased intensity, increased photostability and reduction in radiative lifetime. Metal-enhanced fluorescence (MEF) has been recently used and investigated widely [12–16]. This technique has inspired the present work to use metal nanoparticles to enhance the excitonic emission of graphene. Considering that C–Ni can produce surface plasmon resonances, and Ni is widely used as a catalyst for growing graphene [15], thus the preparation of graphene on nickel nanocrystallites (*nc*-Ni) might be a practical path to enhance its excitonic emission. Si nanoporous pillar arrays (Si-NPAs) have been used as functional substrates for preparing Si-based prototype nanodevices [17–20]. Their rough surface morphology and high reaction activity are beneficial for the nucleation of *nc*-Ni. In this study, we developed an MEF substrate by precipitating *nc*-Ni on Si-NPA and grew graphene nanosheets (GNSs) on it via chemical vapour deposition (CVD). We believe that by combining nanostructured graphene with MEF on a plasmonic nanostructured substrate, strong excitonic emission could be achieved and enable the creation of inexpensive optoelectronic devices.

## 2. Material and methods

### 2.1. Preparation of graphene nanosheets/Si nanoporous pillar array

Si-NPA substrates were prepared by hydrothermally etching (111)-oriented, p-type single crystal Si (*sc*-Si) wafers in a mixed aqueous solution of hydrofluoric acid (13 mol l^−1^) and ferric nitrate (0.04 mol l^−1^) at 140°C for 30 min, as previously described in detail [17]. Subsequently, a layer of *nc*-Ni was deposited on Si-NPA to act as a catalyst for growing GNSs. The *nc*-Ni on Si-NPA was prepared using a chemical bath deposition method. An aqueous solution of 0.2 mol l^−1^ nickel acetate and 4 mol l^−1^ ammonium fluoride was initially prepared, and the pH of the solution was adjusted to 8 using aqua ammonia. Subsequently, the Si-NPA substrate was dipped in the as-prepared solution, and a layer of *nc*-Ni was gradually precipitated on it. The thickness of the Ni layer was controlled by adjusting the soaking time, and the reaction time was set to 20 min in this experiment. With *nc*-Ni as catalyst, GNSs were grown on Si-NPA using CVD. After placing the Ni/Si-NPA in the constant temperature zone of a horizontal tube furnace, the furnace was heated up to 1000°C at a rate of 10°C min^−1^, under the protection of a carrier gas of high-purity H_2_ and Ar (H_2_ : Ar ratio of 65 : 200) with a flow rate of 265 standard cubic centimetre per minute (sccm). Thereafter, this inner temperature of the furnace was held at 1000°C for 20 min and methane gas as carbon source was introduced at a rate of 10 sccm into the furnace with the carrier gas. GNS growth times of 5 and 10 min were used, to prepare GNSs with different thicknesses. The furnace was then cooled down to room temperature at a rate of approximately 10°C s^−1^ under the carrier gas. The samples prepared with 5 and 10 min GNS growth times were labelled A and B, respectively.

### 2.2. Characterization

The surface morphology and structure of the as-prepared GNS/Si-NPA samples were characterized by X-ray diffraction (XRD, Panalytical, X'Pert Pro) with Cu Kα1 radiation (*λ* = 1.5406 Å) as the X-ray source, field-emission scanning electron microscopy (FESEM, JEOL, JSM-6700F), high-resolution transmission electron microscopy (HRTEM, JEOL, JEM-2100) and micro-Raman spectroscopy (Renishaw, RM-2000) using 531 nm laser source and with an incident power of 25 mW. Absorption spectra were recorded on an ultraviolet–visible (UV–vis) spectrophotometer (Shimadzu, UV-3150), using BaSO_4_ powder as a standard for baseline measurements, in the wavelength ranging between 200 and 600 nm. All the above measurements were performed at room temperature (approx. 25°C). The temperature dependence of PL was measured using a double-grating spectrofluorometer (HORIBA, FL-322), which was equipped with a closed-cycle helium cryostat (Janis CCS-100) and a digital temperature controller (LakeShore-325).

## 3. Results and discussion

**Figure 1.**
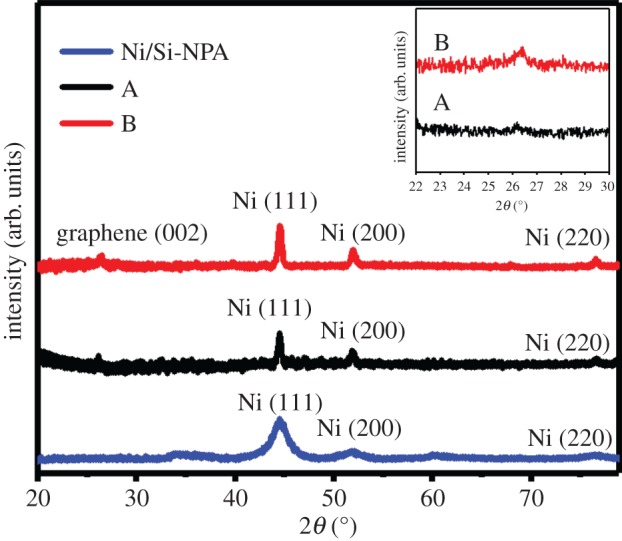
XRD measurements from the Ni/Si-NPA substrate, GNS with 5 min growth time (sample A) and GNS with 10 min growth time (sample B).

The bottom curve in figure 1 presents the XRD pattern of Si-NPA after Ni deposition (Ni/Si-NPA). Apart from the diffraction peaks that appear at 35.8° and 60.9° that originate from the $\left(\bar{1}32\right)$ and (311) family planes of residual C_4_H_14_NiO_8_ (JCPDS No. 24-1360), three diffraction peaks centred at 44.6°, 52.0° and 76.7° are also observed, which are indexed to the diffraction from the (111), (200) and (220) family planes of cubic Ni (JCPDS No. 04-0850), respectively. This finding indicated that Ni had been deposited on Si-NPA after the chemical bath deposition. The XRD pattern of sample A is shown as the middle curve in figure 1. Besides the diffraction peaks of cubic Ni, a broader weak diffraction peak located at 26.2° appears, which can be indexed to the diffraction from the (002) reflection planes of graphene [21]. Moreover, the diffraction peaks for Ni become sharper, which indicated the crystallinity improvement of the Ni film after exposuring to the high temperature in the GNS growth process. For the XRD pattern of sample B (the top curve in figure 1), it is observed that the diffraction peak of graphene became slightly stronger than that in sample A, which indicated the increment of graphene quantity. Meanwhile, the diffraction peak at approximately 26.2° shifted to 26.4°, as shown in the inset of figure 1. The upshift of the peak position implied the decreased interplanar distance of the (002) family planes, which was due to the stronger van der Waals attraction of increment layer of GNSs in sample B. These results implied that graphene had been deposited on Si-NPA after the CVD reaction in samples A and B.

**Figure 2.**
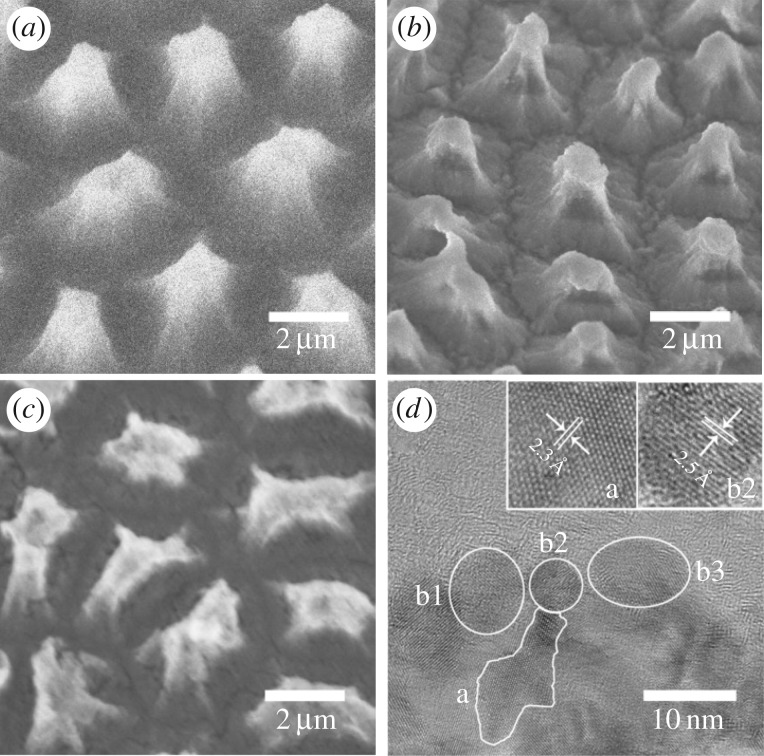
Structural characterization of GNS and the substrate: FESEM image of Si-NPA (*a*), Ni/Si-NPA (*b*), sample A (*c*) by tilting the sample with an angle of 45°. (*d*) HRTEM image of the upper layer cleaved from sample A (insets: magnified images of region a and b2).

FESEM images of the typical surface morphology of Si-NPA, Ni/Si-NPA and sample A are presented in figure 2*a–c* by tilting the sample at an angle of 45°. The Si-NPA consists of a regular array of well-separated Si pillars. The pillars were determined to be perpendicular to the substrate with an average separation distance of approximately 3 μm, and composed of large quantities of Si nanocrystallites enwrapped by SiO_2_, as characterized previously [17]. For the Ni/Si-NPA shown in figure 2*b*, the pillar arrays maintained their original appearance, and became taller and sharper after CBD growth. This condition indicated that the *nc*-Ni grew on the pillar, and preferentially on the top, which agreed with the XRD data. Compared with Ni/Si-NPA, the pillars of sample A maintained their shapes and became slightly shorter after CVD growth, as shown in figure 2*c*. The change of the texture and morphology might be due to the recrystallization and size growth of *nc*-Ni at 1000°C during the CVD process, which was a considerably higher temperature than the highest reported stability temperature of Ni nanograins at 900°C [22,23]. The recrystallization and growth size of *nc*-Ni also corresponded to the narrower full width at half maximum of the Ni XRD peaks in sample A. The upper layer material of sample A was cleaved and the finer structure was characterized by HRTEM, the result of which is shown in figure 2*d*. Large zones with crystal lattices were observed. As shown at the bottom of figure 2*d* (note a), the lattice distance was measured to be 2.3 Å, which corresponded to the expected spacing of (100) family planes of graphene. Lattice spacing was slightly larger than that of graphite (d100 = 0.21 nm) (JCPDS No. 01-1061). As the sample was not oriented at 90° with respect to the electron beam of the TEM, a slight error occurred between the measured and standard expected values. The lattice space of the b region (including b1, b2, b3) was measured to be 2.5 Å and was indexed to the spacing of the cubic Ni (110) planes (JCPDS No. 04-0850). This characteristic confirmed the crystalline nature of GNSs and the typical lateral sizes of the GNS crystallite and *nc*-Ni were approximately 15 × 8 nm and 8 nm, respectively.

**Figure 3.**
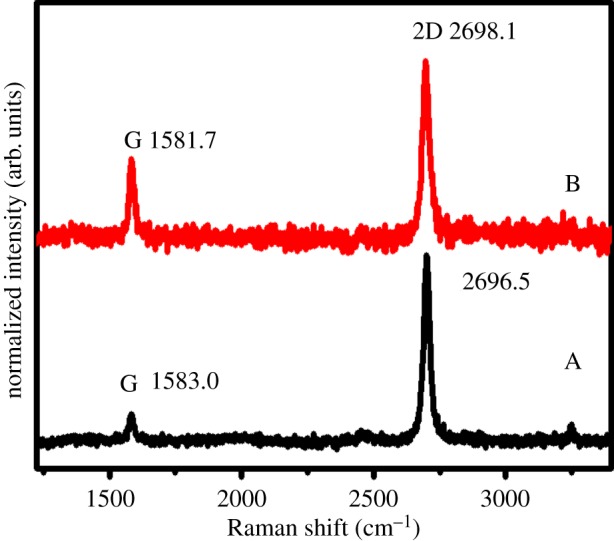
Raman spectra from samples A and B.

Raman spectra can provide detailed information on the quality, stacking order, thickness and even uniformity of graphene [24]. The Raman spectra of samples A and B were measured, and the results are shown in figure 3. The two most prominent features were the G peak at approximately 1583 cm^−1^ and 2D band at approximately 2700 cm^−1^ [25]. The D peak (approx. 1330 cm^−1^), which is due to the breathing modes of six-atom rings and requires a defect for its activation, thereby relating to the defect level in graphene, is missing in figure 3. This finding indicated the overall high quality of the resulting GNSs in samples A and B. The G-band corresponds to the optical mode vibration of two neighbouring carbon atoms in the graphene layer, which was found in sample A at 1583.0 cm^−1^ but shifted down to 1581.7 cm^−1^ in sample B. The downshift of *ω*_G_ followed a 1/*n* dependence on the number of layers *n* without an obvious change in lineshape [26]. For few-layer-thick graphene samples, the relationship can be written as an empirical formula [27]:

3.1 $$\omega_{G}(n)=\omega_{G}(\infty )+\frac{\beta }{n}\;\beta \approx 5.5\;\text{c}{\text{m}}^{-1},$$

where *ω*_G_(*n*) is the wave number of few-layer-thick graphene samples, *ω*_G_(∞) the wavenumber of graphite and *n* the number of graphene layers. We measured the wave number of the G peak of graphite at 1581.0 cm^−1^ by laser spectroscopy at 531 nm. For *ω*_G_(∞) at 1581.0 cm^−1^, we obtained *n* ≈ 3 for sample A and n≈8 for sample B. The spectra of the two specimens were scaled to have similar 2D peak heights at approximately 2700 cm^−1^. A significant change was observed in the peak intensity ratio of 2D to G in different specimens. With reaction time of 5 min for sample A, the 2D peak intensity was roughly six times more than that of the G peak. However, a weaker 2D peak relative to the G peak (roughly two times) was observed with growing time of 10 min for sample B. The 2D peak of sample A was located at 2696.5 cm^−1^, which was slightly downshifted than sample B at 2698.1 cm^−1^. Notably, further increasing the growth time resulted in thicker layers of GNSs, which was also supported by the XRD results [26].

**Figure 4.**
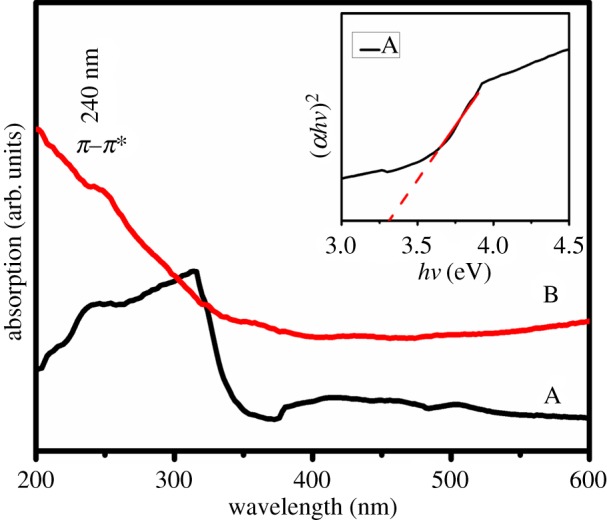
Absorption spectra from samples A and B. Inset: bandgap plot of sample A. The dashed lines at 3.3 eV (376 nm) mark the leading edge of the σ transition.

The optical properties of the GNSs were investigated using highly fluorescent carbon quantum dots as a model. The UV–vis absorption of the two samples was investigated, as shown in figure 4. A pronounced absorption peak, which is typical for GQDs and GO [4,5], occurred at approximately 240 nm, which is related to the π–π* transition of C=C aromatic *sp*^2^ domains. In addition, a new absorption band edge at approximately 370 nm was observed in sample A, whereas sample B showed a broadband absorption ranging from 200 to 600 nm without an obvious absorption band edge, which suggested that the emission of sample A was from band-edge exciton-state decay rather than defect-state decay, as reported previously [5]. The Kubelka–Munk formula and Tauc equation were adopted to determine the absorption band of sample A by using a direct bandgap model. A graph of (*αhν*)^2^ versus *hν* is shown in the inset of figure 4. The absorbed band was calculated to be 3.3 eV.

**Figure 5.**
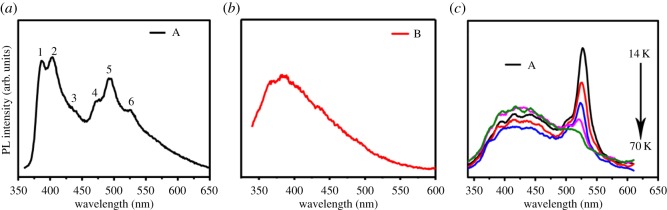
Room-temperature PL from sample A (*a*) and sample B (*b*) with a 320 nm excitation. (*c*) Temperature-dependent PL spectra of sample A from 14 to 70 K at the 320 nm excitation wavelength.

**Figure 6.**
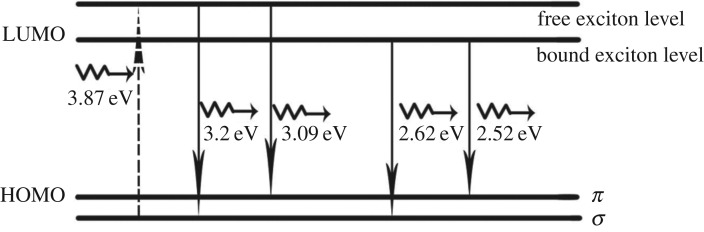
Typical electronic transition schematics of free and bound excitonic recombination observed in the PL spectra of sample A in figure 5*a*.

The most interesting phenomenon found was that several strong PL emission peaks associated with phonon replicas were clearly observed in the PL spectrum of sample A with 320 nm photoexcitation at room temperature, as shown in figure 5*a*. By contrast, the phonon replicas were indistinct for sample B (figure 5*b*). Because GNSs were grown by CVD at high temperature (1000°C) and in a reducing gas environment of Ar and H_2_, PL should originate from the deposited GNSs instead of other functional molecules [4]. Considering the strong electronic screening effect in bulk semiconductors, which decreases the binding energy to only a few tens of millielectronvolt, strong excitonic emission could be only observed in lamina materials with large binding energies [6,28–31]. Therefore, excitonic emission associated with phonon replicas could be observed in few-layer-thick GNSs (sample A) at room temperature, whereas it was extremely weak in multilayer-thick GNSs (sample B). For the energy difference between the absorption band edge (3.3 eV) and PL peak energy, a Stokes shift of 0.1 eV existed, and excitonic emission might originate from the coupling between the excited electrons and lattice phonons. In order to clarify the influence of phonons on the excitonic emission, temperature-dependent PL spectra were measured in the range of 14–70 K, as depicted in figure 5*c*. Under ultraviolet irradiation at 320 nm, two broad emission bands located at approximately 525 and 425 nm were found. The emission peak showed a regular dependence with temperature, in which the peak located at 525 nm exhibited intensity attenuation and the peak intensity of emission peak at 425 nm inversely became strong. This effect can be primarily attributed to the change in the thermal occupation of bound and free excitons [29,32,33]. Thus, given the temperature evolution of the PL spectrum, the peaks at approximately 525 and 425 nm should be attributed to bound and free excitonic emission, respectively. The carbon ground-state multiplicity was related to the energy difference (*E*_g_) between the *σ* and *π* orbitals [4,34]. Aside from the *E*_g_ influence, the excitonic binding energy in the PL spectra should also be taken into consideration, as discussed previously. As shown in figure 5*a*, PL from few-layer-thick GNSs (sample A) obviously came from two mechanisms: free and bound excitonic emission. Therefore, the electronic transitions of approximately 387 nm (3.2 eV, peak 1) and approximately 402 nm (3.08 eV, peak 2) peaks observed in the PL spectra could be regarded as the transition from the lowest unoccupied molecular orbital (LUMO) of free exciton to the highest occupied molecular orbital (HOMO) of *σ* and *π* orbitals. The peaks located at approximately 473 nm (2.62 eV, peak 4) and 492 nm (2.52 eV, peak 5) should belong to the transition from the LUMO of the bound exciton to the HOMO of *σ* and *π* states. Generally, the phonon replica is incidental to the bound excitonic emission in wide bandgap semiconductors. Thus, we attributed the two peaks centred at approximately 439 nm (2.93 eV, peak 3) and 525 nm (2.53 eV, peak 6) to the phonon replica. The recombination process and mechanism of free and bound exciton are demonstrated schematically in figure 6. Based on the energy difference between peaks 1 and 2, *E*_g_ was determined to be approximately 0.1 eV, which satisfies the criterion given by Hoffmann (i.e. smaller than 1.5 eV) [4]. In addition to the effect of the nanostructure of GNSs/Si-NPA, the excitonic emission might also depend on the Ni/Si-NPA substrate. When irradiated by light, *nc*-Ni grown on Si-NPA might generate surface plasmons that amplified the near-field strength. The excitons of GNSs strongly interacted with these surface plasmons, and enabled the excitonic emission of GNSs to be detected at room temperature.

## 4. Conclusion

GNS/Si-NPA samples were prepared via growing GNSs on Si-NPA by CVD. The GNSs obtained in this study were both high quality and well dispersed. Excitonic absorption bands and excitonic emission in the UV–vis region (2.06–3.60 eV) with phonon replicas were observed at room temperature. When combined with the low-temperature emission spectra, we showed that the PL was associated with free and bound excitonic recombination and determined the bandgap of *σ–π* to be approximately 0.1 eV. We believe that the nanostructured graphene and MEF from the Ni/Si-NPA substrate worked together to produce and enhance the fluorescence from the GNSs, which made the excitonic luminescence observable at room temperature. The results presented would be valuable in investigating the origin of the optoelectronic properties of GNSs and expand the potential applications of graphene-based materials to other fields such as optoelectronics.

## Data accessibility

There are no additional data to accompany this manuscript. All relevant datasets are within the main body of the manuscript. All protocols and software used are stated fully in the Material and methods section.
